# Associated factors of neonatal near miss among newborns of adolescent mothers in Brazil

**DOI:** 10.1590/1980-220X-REEUSP-2021-0359en

**Published:** 2022-05-30

**Authors:** Thamara de Souza Campos Assis, Katrini Guidolini Martinelli, Silvana Granado Nogueira da Gama, Edson Theodoro dos Santos

**Affiliations:** 1 Universidade Federal do Espírito Santo, Departamento de Medicina Social, Programa de Pós-graduação em Saúde Coletiva, Vitória, ES, Brazil.; 2 Fundação Oswaldo Cruz, Escola Nacional de Saúde Pública Sérgio Arouca, Rio de Janeiro, RJ, Brazil.

**Keywords:** Near Miss, Healthcare, Pregnancy Complications, Pregnancy in Adolescence, Prenatal Care, Maternal-Child Health Services, Near Miss Salud, Complicaciones del Embarazo, Embarazo en Adolescencia, Atención Prenatal, Servicios de Salud Materno-Infantil, Near miss, Complicações na Gravidez, Gravidez na Adolescência, Cuidado Pré-Natal, Serviços de Saúde Materno-Infantil

## Abstract

**Objective:**

To identify the associated factors of neonatal near miss among newborns of Brazilian adolescents and to compare their occurrence in young women aged 12 to 16 years and 17 to 19 years.

**Method:**

Cross-sectional, hospital-based study, using data from the study *Nascer no Brasil* (“Birth in Brazil”) on puerperal adolescents and their newborns in all regions of Brazil. Multiple and univariate logistic regression were employed to identify the associated factors of neonatal near miss.

**Results:**

The following factors were found to be associated to neonatal near miss among newborns of adolescent mothers: public source of payment (OR = 4.57, 95% CI = 2.02–10.32), having to seek help in different maternity hospitals (OR = 1.52; 95% CI = 1.05–2.20), and maternal near miss (OR = 5.92; 95% CI = 1.94–18.05), in addition to a record of low weight in a previous pregnancy (OR = 3.12; 95% CI = 1.61–6.04) and twin pregnancy (OR = 7.49; 95% CI = 3.28–16.82).

**Conclusion:**

Neonatal near miss affected newborns of adolescent mothers in both age groups equally. Also, the determinant factors of neonatal near miss can be mostly reduced with qualified prenatal, labor, and birth care.

## RESUMO

**Objetivo:**

Identificar os fatores associados ao *near miss* neonatal em recém-nascidos de adolescentes brasileiras e comparar sua ocorrência entre as jovens de 12 a 16 anos e as de 17 a 19 anos.

**Método:**

Estudo transversal, de base hospitalar, com dados da pesquisa “Nascer no Brasil”, composto por puérperas adolescentes e seus recém-nascidos em todas as regiões do país. Utilizou-se regressão logística univariada e múltipla para identificar os fatores associados ao *near miss* neonatal.

**Resultados:**

Mostraram-se associados ao *near miss* neonatal de recém-nascidos de mães adolescentes os fatores fonte de pagamento público (OR = 4,57, IC95% = 2,02–10,32), peregrinação por maternidades (OR = 1,52; IC95% = 1,05–2,20) e presença de *near miss* materno (OR = 5,92; IC95% = 1,94–18,05), além de histórico de baixo peso em gestação anterior (OR = 3,12; IC95% = 1,61–6,04) e gemelaridade (OR = 7,49; IC95% = 3,28–16,82).

**Conclusão:**

O *near miss* neonatal acometeu igualmente os recém-nascidos de mães adolescentes de ambas as faixas etárias. Além disso, os determinantes do *near miss* neonatal, em sua maioria, podem ser reduzidos com atenção qualificada ao pré-natal, parto e nascimento.

## RESUMEN

**Objetivo:**

Identificar los factores asociados al *near miss* neonatal en recién nacidos de adolescentes brasileñas y comparar su ocurrencia entre las jóvenes de 12 a 16 años y las de 17 a 19 años.

**Método:**

Estudio transversal, de base hospitalaria, con datos de la investigación *Nascer no Brasil*, compuesta por puérperas adolescentes y sus recién nacidos en todas las regiones de Brasil. Se utilizó regresión logística univariante y múltiple para identificar los factores asociados al *near miss* neonatal.

**Resultados:**

Se asociaron al *near miss* neonatal los factores fuente de pago pública (OR = 4,57, IC95% = 2,02–10,32), peregrinación por hospitales de maternidad (OR = 1,52; CI95% = 1,05–2,20) y la presencia de *near miss* materno (OR = 5,92; CI95% = 1,94–18,05), además de los antecedentes de bajo peso en el embarazo anterior (OR = 3,12; CI95% = 1,61–6,04) y el embarazo gemelar (OR = 7,49; CI95% = 3,28–16,82).

**Conclusión:**

El *near miss* neonatal afectó igualmente a los recién nacidos de madres adolescentes de ambos grupos de edad. Además, los factores determinantes del *near miss* neonatal, en su mayoría, pueden reducirse con la atención cualificada al prenatal, al parto y al nacimiento.

## INTRODUCTION

Adolescent pregnancy is common in unequal and socioeconomically unfavorable settings^(1)^. Adolescents usually start prenatal care later and attend fewer consultations^(2,3)^, which makes them more vulnerable to negative neonatal outcomes^(4)^; this has been observed in Brazil and several other countries^(5)^.

Neonatal near miss is a severity indicator defined as near death of a newborn after surviving for 27 days^(6)^. According to a study by Lima et al, newborns of adolescent mothers have a 60% higher risk of neonatal near miss when compared to adult mothers^(7)^. In addition, maternal age under 20 years has been associated to very low birth weight (<1,500 g) and severe or extreme prematurity (<32 weeks), both of which compose neonatal near miss^(8,9)^.

The rates of neonatal near miss are estimated to be four to six times higher than mortality in the same age group. Therefore, classifying neonatal near miss is a means to increase the study’s power to detect risk factors associated to death^(10)^. The hypothesis is that this outcome is more frequent in pregnancies of younger adolescents.

Neonatal near miss as an indicator became more widely employed from the 2000s onwards; thus, epidemiological studies dealing with this issue in adolescence are still scarce due to requiring significant samples with a population coverage that includes adolescent mothers of different ages and their newborns^(11)^. The objective of this article is therefore to identify the associated factors of neonatal near miss in newborns of Brazilian adolescents and to compare its occurrence among young women aged 12 to 16 years and 17 to 19 years.

## METHOD

### Design of Study

This is a cross-sectional, hospital-based study, whose data were obtained from the Brazilian study *Nascer no Brasil* (“Birth in Brazil”).

### Local

This study included puerperal women and their newborns, whose data were collected from February 2011 to October 2012, and was conducted in three stages: the first one included hospitals with over 500 births/year, which were stratified by the Brazilian macroregions (North, South, Northeast, Southeast, and Center-West), location (capital or inland), and type of service in which delivery took place (public, private, or mixed); in the second stage, the number of required days to interview 90 puerperal women in each of the 266 previously selected hospitals (a minimum of seven days) was defined through the inverse sampling method; and finally, in the third stage, puerperal women and their conceptuses were selected^(12)^.

### Population

The study *Nascer no Brasil* interviewed 23,894 puerperal women of all age groups. However, for this analysis, only puerperal adolescents and their newborns were considered, totaling a sample of 4,571 puerperal women (approximately 20%) under 20 years old, who were categorized into 12 to 16 or 17 to 19 years old. This cutpoint was defined due to some studies stating that women over sixteen years old have an obstetric performance similar to that of adult women^(2,13)^. No puerperal females under 12 years old were found in this study.

### Data Collection

The data were collected through electronic forms. This study included information from the following sources: interview with the puerperal women during hospitalization; photographed and transcribed prenatal cards; mothers and newborns’ medical records. The latter were collected after the females were discharged or on the 42nd day of hospitalization and/or after discharge or on the 28th day of newborn hospitalization^(14)^.

### Data Analysis and Treatment

The variable neonatal near miss was elaborated accounting for two surveys conducted by the World Health Organization (WHO)^(15)^. The presence of any of the following characteristics indicated neonatal near miss. Pragmatic criteria: Apgar Index <7 at 5 minutes, weight at birth <1,750 grams, and gestational age ≤32 weeks. Management criteria: using antibiotics, continuous positive airway pressure (CPAP), phototherapy in the first 72 hours, vasoactive drug, anticonvulsants, surfactants, receiving cardiac massage, hypoglycemia, and orotracheal intubation.

The following sociodemographic variables were used: mother’s age (12–16 years old, 17–19 years old), pregestational Body Mass Index (BMI) – Kg/m2 (<18.5 = underweight; 18.5–24 = normal; 25.0–29.9 = overweight; 30.0 or more = obese), economic classification by the Brazilian Market Research Institutes Association (classes A/B, C, D/E), race/color (white, black, brown, Asian, and Indigenous) and area of residence (North, Northeast, Southeast, South, and Center-West). For an analysis of maternal risk behaviors, suspicion of alcohol misuse was included (“yes”, when the female had a score of two or more out of seven in the instrument *Tolerance Worry Eye-opener Annoyed Cut-down* (TWEAK), or “no”, when she drank no alcohol during pregnancy or had a score of one)^(16)^, as well as whether females had smoked during the pregnancy (yes or no).

The variable related to the minimum overall adequacy of prenatal was adapted from the prenatal care adequacy recommended by the Brazilian Ministry of Health and was classified as adequate or inadequate^(3)^. Prenatal was considered to be minimally adequate when: care started up to the 12th week of gestation; the number of consultations was adequate to the gestation age at birth (one at three months, two at six, and three consultations at nine months), with at least one of the following routine tests: syphilis serology, fasting glycemia, urine, HIV serology, and ultrasound; and mother’s report on orientation for reference hospital^(3)^.

The included obstetric records were previous abortion, history of prematurity, and history of low weight in a previous gestation, all of which were classified as present or absent. When accounting for delivery, the following variables were used: source of payment for the delivery (public, private), having to seek help in different maternity hospitals – unable to receive care in the first maternity approached for the delivery (yes/no), type of delivery (vaginal or forceps, caesarean cut) and maternal near miss (yes/no). Pre-gestational diabetes was included among pre-gestational diseases. Pregnancy complications included: hypertensive disease (chronic hypertension, preeclampsia, eclampsia, or HELLP syndrome), gestational diabetes, urinary infection, and syphilis, all of which were classified as present or absent. Presence or absence of multiple gestation was also considered. Maternal near miss was classified according to clinical, laboratory, and management criteria which were defined and consolidated by the WHO^(17)^.

The complex sampling design was considered throughout the statistical analysis. Each selected stratum was calibrated by the ratios of basic sample weights to ensure that the distribution of puerperal women was like that of births of the sampled population in 2011, deriving weighted percentages.

Chi-squared test was employed (χ^2^) to verify differences among proportions, considering a 95% confidence interval (95% CI). Subsequently, to verify the characteristics of neonatal near miss to which maternal age was associated, univariate and multiple logistic regression was conducted. The effect of the interaction was tested before the final analysis during regression. The pseudo-R^2^ (Cox & Snell e Nagelkerke) statistic was used to choose the best adjustment for the model, whose value was closer to 1. The adjusted analyses included all variables of the unadjusted analysis with p-value < 0.20.

### Ethical Aspects

This study was approved by the Research Ethics Committee of the National Public Health School of Fundação Oswaldo Cruz on opinion n. 92/2010. Digital consent was obtained from each puerperal adolescent and their guardians after reading the Informed Consent Form before the interview. The secondary data analysis performed in this article was approved by the Research Ethics Committee of the Health Sciences Center of Universidade Federal do Espírito Santo on opinion. 3.565.689/2019.

## RESULTS

This study’s participants amounted to 4,541 puerperal adolescents, 1,356 of whom were aged 12 to 16 years, whereas 3,185 adolescents were aged 17 to 19 years. Neonatal near miss and its indicators presented no statistically significant differences between women aged 12 to 16 years and those aged 17 to 19 years (Table 1).

**Table 1.** Indicators composing neonatal near miss – Brazil, 2011–2012.

Variables	Maternal age		
	12–16 years old (1,356)	17–19 years old (3,185)	χ2
	n (%)	n (%)	P-value
Neonatal near miss indicator			
No	1,219 (90.5)	2,878 (90.7)	0.075
Yes	128 (9.5)	295 (9.3)	
Neonatal Near Miss			
Apgar <7 at 5 min.	11 (0.8)	21 (0.7)	0.709
Weight <1,750 g	27 (2.0)	54 (1.7)	0.556
Gestational Age ≤32 weeks	35 (2.6)	121 (3.8)	0.115
Vasoactive drugs	09 (0.7)	33 (1.0)	0.326
Nasal CPAP	24 (1.8)	65 (2.1)	0.637
Antibiotics	35 (2.6)	121 (3.8)	0.115
Intubation	25 (1.9)	52 (1.6)	0.702
Phototherapy	55 (4.1)	127 (4.0)	0.974
Cardiac massage	10 (0.7)	19 (0.6)	0.656
Anticonvulsant	02 (0.1)	06 (0.2)	0.928
Surfactant	19 (1.4)	32 (1.0)	0.391
Hypoglycemia	12 (0.9)	41 (1.3)	0.427

Out of the 128 younger adolescents (12–16 years old) whose newborns were classified with neonatal near miss, 78 (61.4%) presented one characteristic which configured this outcome, 15 (11.8%) two characteristics, 11 (8.7%) three characteristics, and 23 (18.1%) four or more characteristics. Out of 295 older adolescents (17–19 years old), 152 (51.4%) presented one characteristic, 68 (23.0%) two characteristics, 21 (7.1%) three characteristics, and 55 (18.5%) four or more characteristics which comprise neonatal near miss.

Table 2 shows factors associated to neonatal near miss in accordance with maternal age and socioeconomic, prenatal, and delivery characteristics and risk behavior; the statistically significant differences for characteristics of prenatal and delivery were inadequate prenatal (p = 0.061), public source of payment (p < 0.001), and having to seek help in different maternity hospitals (p = 0.008).

**Table 2.** Associated factors of neonatal near miss according to maternal age, socioeconomic, prenatal, and delivery characteristics, and risk behavior – Brazil, 2011–2012.

Variables	Neonatal Near Miss		
	YES (423)	NO (4,097)	χ2
	n (%)	n (%)	P-value
**Socioeconomic characteristics**			
Maternal age			0.930
12–16 years old	128 (9.5)	1,219 (90.5)	
17–19 years old	295 (10.3)	2,878 (89.7)	
Marital status*			0.896
No partner	135 (9.5)	1,284 (90.5)	
Partnered	288 (9.3)	2,809 (90.7)	
Economic classification*			0.513
Class D+E	157 (10.1)	1,394 (89.9)	
Class C	227 (9.3)	2,220 (90.7)	
Class A+B	37 (8.2)	450 (91.8)	
Skin color			0.245
White	98 (7.9)	1,139 (92.1)	
Black	27 (6.9)	365 (93.1)	
Brown	290 (10.3)	2,531 (89.7)	
Asian	5 (11.4)	39 (88.6)	
Indigenous	3 (9.7)	28 (90.3)	
Region			0.648
North	47 (7.8)	554 (92.2)	
Northeast	160 (11.1)	1.284 (88.9)	
Southeast	144 (8.6)	1,534 (91.4)	
South	48 (9.8)	440 (90.2)	
Center-West	24 (7.8)	284 (92.2)	
Pre-gestational BMI (Kg/m^2^)*			0.572
Underweight	74 (10.9)	607 (89.1)	
Normal	276 (8.8)	2,866 (91.2)	
Overweight	60 (10.6)	506 (89.4)	
Obese	13 (11.0)	118 (89.0)	
**Risk behavior**			
Alcohol use during pregnancy*			0.972
Yes	34 (9.1)	338 (90.9)	
No	373 (10.2)	3,657 (89.8)	
Smoking during pregnancy*			0.773
Half of the pregnancy	08 (8.9)	82 (91.1)	
Whole pregnancy	18 (10.8)	149 (89.2)	
No	397 (9.3)	3,865 (90.7)	
**Prenatal and delivery characteristics**			
Prenatal adequacy			**0.061**
Adequate	35 (6.8)	483 (13.2)	
Inadequate	388 (9.7)	3,614 (90.3)	
Source of payment of the delivery*			**0.000**
Public	417 (9.8)	3,855 (90.2)	
Private	05 (2.0)	241 (98.0)	
Sought help in different maternity hospitals*			**0.008**
Yes	151 (12.9)	1,018 (87.1)	
No	272 (8.1)	3,072 (91.9)	
Type of delivery			0.571
Vaginal/forceps	255 (9.0)	2,565 (91.0)	
Caesarean section	168 (9.9)	1,532 (90.1)	

*Missing data

Table 3 presents the associated factors of neonatal near miss; multiple pregnancy (p < 0.001), maternal near miss (p < 0.001), hypertensive disease (p = 0.027) and a record of prematurity (p = 0.023) and low weight at birth (p = 0.004) were characteristics of the current and previous pregnancy which were associated to neonatal near miss in puerperal adolescents.

**Table 3.** Factors associated to neonatal near miss according to characteristics of the current pregnancy, complications, prepregnancy diseases, and obstetric record – Brazil, 2011**–**2012.

Variables	Neonatal Near Miss		
	YES (423)	NO (4,097)	χ2
	n (%)	n (%)	P-value
Parity*			0.533
Primiparous	353 (9.6)	3,326 (90.4)	
Multiparous	70 (8.3)	770 (91.7)	
Multiple pregnancies			**0.000**
Yes	25 (40.3)	37 (59.7)	
No	398 (8.9)	4,060 (91.1)	
Maternal Near Miss*			**0.000**
Yes	16 (39.0)	25 (61.0)	
No	407 (9.1)	4,071 (90.9)	
Hypertensive disease#			**0.027**
Yes	51 (13.6)	325 (86.4)	
No	372 (9.0)	3,772 (91.0)	
Gestational Diabetes*			0.284
Yes	14 (6.9)	190 (93.1)	
No	409 (9.5)	3,906 (90.5)	
Pregestational diabetes			0.412
Yes	04 (15.4)	22 (84.6)	
No	419 (9.3)	4,075 (90.7)	
Urinary infection*			0.777
Yes	179 (9.4)	1,722 (90.6)	
No	232 (9.0)	2,351 (91.0)	
Syphilis*			**0.051**
Yes	08 (20.5)	31 (79.5)	
No	401 (9.0)	4,049 (91.0)	
Previous abortion*			0.168
Yes	46 (13.1)	306 (86.9)	
No	67 (8.6)	714 (91.4)	
Primigravida	309 (9.1)	3,077 (90.9)	
Prematurity Record*			**0.023**
Yes	15 (17.0)	73 (83.0)	
No	54 (7.2)	698 (92.8)	
Primigravida	353 (9.6)	3,326 (90.4)	
Low Weight Record*			**0.004**
Yes	20 (18.5)	88 (81.5)	
No	50 (6.8)	682 (93.2)	
Primigravida	353 (9.6)	3,326 (90.4)	

# Hypertensive disease = chronic hypertension, preeclampsia, eclampsia, and HEELP syndrome * Missing data

Regardless of the maternal age group, after the first variable adjustment, a higher chance of neonatal near miss was found for prenatal inadequacy (OR = 1.60, 95% CI = 1.02–2.51) and, in multiple analysis, public source of payment of delivery (OR = 4.57, 95% CI = 2.02–10.32), having to seek help in different maternity hospitals (OR = 1.52; 95% CI = 1.05–2.20), multiple pregnancy (OR = 7.42; 95% CI = 3.28–16.82), presence of maternal near miss (OR = 5.92; 95% CI = 1.94–18.05), and a history of low birth weight (OR = 3.12; 95% CI = 1.61–6.04) (Table 4).

**Table 4.** Multiple logistic regression model of factors associated to neonatal near miss in newborns of adolescent mothers – Brazil, 2011–2012.

Variables	Neonatal Near Miss		
	Yes		
	Raw OR (95% CI)	Adjusted OR^a^ (95%CI)	Adjusted OR^b^ (95%CI)
Prenatal adequacy			
Adequate	1.00	1.00	1.00
Inadequate	1.48(0.98 – 2.25)	**1.60(1.02 – 2.51)**	1.49(0.96 – 2.30)
Source of payment of the delivery			
Private	1.00	1.00	1.00
Public	**4.76(2.09 – 10.83)**	**4.27(1.89 – 9.67)**	**4.57(2.02 – 10.32)**
Sought help in different maternity hospitals			
No	1.00	1.00	1.00
Yes	**1.67(1.14 – 2.44)**	**1.49 (1.03 – 2.16)**	**1.52(1.05 – 2.20)**
Multiple pregnancies			
No	1.00	1.00	1.00
Yes	**6.85(3.06 – 15.36)**	**5.15(2.09 – 12.68)**	**7.42(3.28 – 16.82)**
Maternal Near Miss			
No	1.00	1.00	1.00
Yes	**6.22(1.95 – 19.86)**	**5.02(1.63 – 15.45)**	**5.92(1.94 – 18.05)**
Hypertensive disease			
No	1.00	1.00	**–**
Yes	**1.60(1.05 – 2.45)**	1.35(0.89 – 2.02)	**–**
Syphilis			
No	1.00	1.00	**–**
Yes	2.51(0.96 – 6.56)	2.21(0.80 – 6.08)	**–**
Previous abortion			
No	1.00	1.00	**–**
Yes	1.60(0.89 – 2.87)	0.97(0.50 – 1.88)	**–**
Primigravida	1.07(0.75 – 1.53)	0.61(0.32 – 1.16)	**–**
Prematurity record			
No	1.00	1.00	**–**
Yes	**2.69(1.41 – 5.12)**	1.56(0.74 – 3.29)	**–**
Primigravida	1.36(0.92 – 2.01)	0.93(0.37 – 2.31)	**–**
Record of Low birth weight in previous pregnancy			**–**
No	1.00	1.00	1.00
Yes	**3.11(1.66 – 5.86)**	**2.60(1.20 – 5.65)**	**3.12(1.61 – 6.04)**
Primigravida	1.46(0.98 – 2.17)	**2.60(1.20 – 5.65)**	1.41(0.94 – 2.12)

^a^ Model with all variables presenting p-value under 0.20 in the chi-squared test. ^b^ Adjusted model only with variables that remained in the final model. This model presented the best adjustment, since the value of pseudo-R2 (Cox & Snell and Nagelkerke) was closer to 1.

## DISCUSSION

The results of this study point out that 9.5% of the newborns of puerperal adolescents aged 12 to 16 years presented neonatal near miss, similarly to those aged 17–19 years old (9.3%). The factors associated to neonatal near miss were prenatal inadequacy, public source of payment of the delivery, twin pregnancy, having to seek help in different maternity hospitals, and the presence of severe maternal complications during pregnancy and delivery, in addition to a record of low birth weight in a previous pregnancy.

Prenatal inadequacy is one of the contributing factors to negative outcomes in newborns. Brazilian studies have found an association of neonatal near miss with fewer than six consultations (OR = 3.57, 95% CI = 2.57–4.94)^(7)^, fewer than four prenatal consultations (OR = 1.8, 95% CI = 2.0–1.9)^(18)^, and lack of an adequate prenatal (OR = 17.4; 95% CI = 6.5–46.8)^(19)^. In Indonesia, researchers have identified that the fewer the prenatal visits attended by women, the higher the chances of neonatal near miss (OR = 6.70, 95% CI = 2.71–16.62)^(8)^.

Adolescents generally receive more inadequate prenatal care in comparison to adults, with a late start and fewer consultations than recommended by the Brazilian Ministry of Health. This may be attributed to social inequality issues, which hinder the diagnosis of pregnancy, and access barriers to health services^(3,20)^. The difficulty of accepting a generally unintended pregnancy is also an obstacle to an early start of prenatal care^(21)^.

Therefore, the chances of complication are magnified, making the adolescent more vulnerable to diseases which are specific to pregnancy^(20)^ and, consequently, to negative neonatal outcomes, such as neonatal near miss. It is important to emphasize that when health services offer pertinent care and orientation on pregnancy, delivery, and puerperium, a bond of confidence and respect with the adolescent is created and adherence to prenatal follow-up is increased, minimizing thus possible negative outcomes^(20,21)^.

Data from the study *Nascer no Brasil* (2011–2012) shows that private (OR = 0.60; 95% CI: 0.42–0.86) and mixed hospitals (OR = 0.58; 95% CI: 0.41–0.83) present lower chances of neonatal near miss in comparison with public hospitals^(10)^. In Brazil, the Unified Health System (*Sistema Único de Saúde* – SUS) provides public and free care to nearly 80% of the population and owns most of the Neonatal Intensive Care Units (NICU)^(22)^, which makes it impossible to infer a worse quality of healthcare due to a higher neonatal morbidity rate; disease severity is a powerful confounding factor^(23)^.

Adolescent mothers are more exposed to failing to receive care in the first maternity service approached for the delivery, as shown; in this context of social inequality, mothers and their fetuses present a higher risk of death and near miss due to having to seek help in different maternity hospitals. In medium and small size municipalities of the Jequitinhonha Valley (Minas Gerais state) and the Northeast and North regions of Brazil, an association was found between not being associated to a maternity hospital during prenatal and child death (OR = 1.28; 95% CI = 1.02–1.61)^(24)^. In the state of Ceará, Brazil, a study has shown that late access by pregnant adolescents to specialized services leads to neonatal near miss (OR = 3.0; 95% CI = 1.8–5.1) in comparison with adults^(25)^. Regardless of the maternal age group, better perinatal results are obtained in hospitals with adequate material and human resources, in addition to assertive care practices towards pregnant and puerperal women and their newborns^(23)^, suggesting the importance of an association between adolescents and an appropriate hospital to meet their needs.

The search for delivery care in more than one maternity hospital increases the chances of severe maternal complications, particularly in age extremes, i.e., under 15 and over 35 years old^(26)^. In Ethiopia, a study conducted in three major hospitals has shown an association of maternal complications with neonatal near miss (OR = 12.86; 95% CI = 7.8–21.1)^(27)^.

Conditions such as multiple pregnancy, hypertensive disease, and syphilis infection may lead to complications both during prenatal and delivery and to unfavorable neonatal outcomes. In India, neonatal near miss was shown to be associated to a history of hospitalization in the current pregnancy (OR = 2.75; 95% CI = 1.12–6.70)^(28)^. In Brazil, a study conducted in six maternity units of the Southeast region in 2011 has found an association of hypertensive diseases (OR = 3.0; 95% CI: 2.0–4.4) and syphilis infection (OR = 3.3; 95% CI:1.5–7.2) with neonatal near miss^(19)^.

A history of low birth weight in a previous pregnancy (LBW) was associated to neonatal near miss, since multiparous women who have had low weight babies in the first pregnancies are known to present a higher chance of having babies with LBW in subsequent pregnancies; very low birth weight is one of the pragmatic components of neonatal near miss^(29)^. Also, children born from adolescent mothers, when compared to adult mothers, are known to have a lower weight throughout life, as shown by an Indian study with over 60 thousand women^(30)^. In England, a study considered the newborns’ weight and maternal age in the first and second pregnancies; women aged 14 to 17 years, during their second birth, had babies with significantly lower birth weight (adjusted difference = –80 g; 95% CI: –115, –46) when compared to adults^(31)^.

This study is remarkable for using data from the study *Nascer no Brasil*, the first Brazilian study with obstetric and perinatal data, which includes puerperal adolescents, nearly 20% of the nationwide sample, considering deliveries in public, private, and mixed hospitals. In addition, the importance of analyzing the outcome neonatal near miss, so far understudied in Brazil, is emphasized. However, this study presents limitations, such as the time since data collection and the non-inclusion of hospitals with fewer than 500 births per year, which represented 22.9% of births in Brazil.

## CONCLUSION

Adolescent pregnancy, particularly among the youngest adolescents, creates risk for women and their newborns. In this study, neonatal near miss has equally affected newborns of adolescent mothers of both analyzed age groups. The following factors were shown to be associated to neonatal near miss: public source of payment, having to seek help in different maternity hospitals, and presence of near miss, in addition to a history of low weight in a previous pregnancy and twin pregnancy.

Health policies targeted at adolescents are required; these must account for sexual rights, with egalitarian practices aimed at reducing the social and cultural barriers to health education, as well as effective and participative programs. Thus, in addition to preventing unplanned adolescent pregnancy, issues such as violence and sexually transmitted infections might be avoided.

An adequate access to health services and qualified care towards adolescent prenatal and delivery may contribute to avoid unfavorable neonatal outcomes and promote healthy births. It is therefore fundamental that prenatal be offered in accordance with the protocol of the Brazilian Ministry of Health, including tests, orientation, and care adapted to the maternal age group. In addition, an early diagnosis of risk pregnancy, with due referral to specialized care and a relation with a maternity hospital, shall contribute to more favorable results during pregnancy, delivery, and birth, preventing thus neonatal near miss.

## ASSOCIATE EDITOR

Maria Luiza Gonzalez Riesco
